# Spatiotemporal patterns of youth isolation and loneliness in the US: a geospatial analysis of Crisis Text Line data (2016–2022)

**DOI:** 10.1007/s10708-024-11253-w

**Published:** 2024-12-03

**Authors:** Christopher Lucero, Margaret M. Sugg, Sophia C. Ryan, Jennifer D. Runkle, Martie P. Thompson

**Affiliations:** 1 Department of Geography and Planning, Appalachian State University, Boone, NC USA; 2 Department of Geography and Environment, University of North Carolina Chapel Hill, Chapel Hill, NC USA; 3 North Carolina Institute for Climate Studies, North Carolina State University, Asheville, NC USA; 4 Department of Public Health and Exercise Science, Appalachian State University, Boone, NC USA

**Keywords:** COVID-19 pandemic, Loneliness, Isolation, Mental health, SatScan, Geospatial

## Abstract

In 2021, the US Surgeon General issued a national advisory citing an epidemic of isolation and loneliness. Even before the onset of the COVID-19 pandemic, approximately half of people in the US reported experiencing measurable levels of loneliness. Despite localized and select cross-sectional studies highlighting even higher increases in isolation/loneliness during the COVID-19 pandemic, additional research is needed, particularly for youth and young adults. This work examines patterns of isolation/loneliness across the US from 2016 to 2022 among individuals aged 24 and younger. Our study leverages a unique dataset, Crisis Text Line, which provides complete spatiotemporal coverage of crisis conversations in the US. We conducted a geospatial analysis using Kuldroff’s Space–Time SatScan to identify statistically significant clustering of elevated isolation/loneliness-related conversations. The statistical significance of spatiotemporal clusters was determined using Monte Carlo simulations (n = 9999). Results demonstrated local relative risk as high as 1.47 in high-risk populations in Southern, Midwest, and Atlantic states, indicating areas where the actual case count is 147% of the expected cases (*p* value < 0.01) from May to July 2020. Results also identified co-occurrence of isolation/loneliness and other crises concerns, including depression/sadness, anxiety, and multiple suicidality indicators, with higher rates among racial/ethnic minority, transgender and gender diverse, and younger individuals. This work makes a unique contribution to the literature by elucidating spatiotemporal disparities in isolation/loneliness among young people, providing much-needed knowledge as to where future public health interventions are immediately needed.

**Supplementary Information:**

The online version contains supplementary material available at 10.1007/s10708-024-11253-w.

## Introduction

Isolation/loneliness levels have been increasing since 2012, with concerning trends identified for youth and young adults (Twenge et al., 2019). A 2020 survey by the United Health Foundation (UH) found that, of Americans between the ages of 18 and 34, 48% reported feeling isolated (AARP and UHF, 2020). In 2021, a national survey by the Harvard Graduate School of Education found that loneliness, defined as "the negative feelings from a perceived gap between desired and actual relationships," increased significantly due to the COVID-19 pandemic. Among the 950 respondents, 61% of young adults reported experiencing "serious loneliness" (Weissbourd et al., 2021).

This loneliness epidemic was acknowledged by the United States government in 2021 in a national advisory released by the United States Surgeon General (Office of the Surgeon General, 2021). This advisory warns that young people are at a heightened risk of perceiving isolation/loneliness within their own lives, a phenomenon worsened by social distancing and COVID-19 public health restrictions. However, the official declaration of the loneliness epidemic came in May of 2023 when the Surgeon General released an official report entitled *Our Epidemic of Loneliness and Isolation.* In this report, it was noted that although the pandemic was universally experienced, its effects were disproportionately felt by specific demographic groups (Office of the Surgeon General, 2023). Specifically, youth and adolescents are particularly vulnerable, with one study reporting that people aged 18 to 24 are almost twice as likely (79%) to report feeling lonely than older adults over 65 years old (41%) (The Cigna Group, 2022).

Emerging research from the COVID-19 pandemic reflects higher mental health burdens among youth (Runkle et al., 2023; Groarke et al., 2020) and young adults (Bu et al., 2020; Losada-Baltar et al., 2020). Specifically, significant increases in anxious and depressive symptomology have been identified during the COVID-19 pandemic among these age groups (Bell et al., 2023; Racine et al., 2021; Ravens-Sieberer et al., 2022; Temple et al., 2022). Isolation was consistently identified as a significant upstream contributing factor to the increases in anxiety and depression (Bell et al., 2023; Racine et al., 2021; Temple et al., 2022). A previous study into the effects of loneliness and social isolation on youth mental and physical health using self-reported cross-sectional data found a strong association between isolation and depression, anxiety, substance use, and suicide (Christiansen et al., 2021).

Our study seeks to extend to the existing knowledge base by examining the prevalence of isolation/loneliness clusters in space and time during the COVID-19 pandemic. This study explores several key questions regarding isolation/loneliness among young people in the contiguous United States between 2016 and 2022. First, it examines the spatiotemporal trends in isolation/loneliness during this period, identifying geographic and temporal patterns. Secondly, the research investigates whether the demographic characteristics of individuals seeking help for isolation/loneliness differ from those seeking assistance for other issues, such as mental health concerns, relationship problems, or substance use. Additionally, the study assesses how consistent these patterns are across different racial groups, age groups, genders, and stages of the COVID-19 pandemic. Finally, the research explores the co-occurrence of isolation/loneliness with other crises, identifying how frequently these issues overlap and which populations are most at risk for experiencing multiple crises. The results of this study provide new evidence on the youth populations most vulnerable to isolation/loneliness.

## Methodology

### Data

We used data from Crisis Text Line (CTL), a nonprofit text-based crisis counseling service, on crisis conversations between CTL counselors and individuals in the United States from January 2016 to December 2022. Counselors code the conversation based on the crises of concern (e.g., stress/anxiety, depression/sadness, and thoughts of suicide), and the individuals in crisis are asked to complete an anonymous demographic survey. The demographic survey provides information on age, race, gender identity, and sexual orientation, a unique strength of this dataset, given that subgroup information, notably gender and sexual identity, are often missing in large health datasets (National Institute of Health, 2019). CTL data was analyzed in two separate datasets: (1) all crisis conversations and (2) data restricted to individuals who had completed the demographic survey and provided their age as 24 or younger (13 or younger, 14–17, or 18–24), and had a telephone area code located in the contiguous US. Between 2016 and 2022, there were 183,379 conversations hand-tagged by crisis counselors as being about ‘isolation/loneliness’ among youth and young adult texters under 25. To further analyze these trends throughout the pandemic, we subsetted the isolation/loneliness repository into three separate periods: (1) The Pre-COVID-19 time period, defined as the start of the dataset (January 2016) through February 2020; (2) The Early COVID-19 time period defined as March 2020, when the federal government declared a State of Emergency (Federal Emergency Management Agency, 2020), through April 2021; and (3) the Late COVID-19 time period, defined as May 2021–December 2022, as that is when the Federal Drug Administration authorized the administration of COVID-19 vaccinations to children between the ages of 12 and 15 (Assistant Secretary for Public Affairs, 2024). Data was arranged as a monthly time series depicting the number of isolation/loneliness conversations and total conversations per area code per month.

### SaTScan

For our spatiotemporal analysis, we employed the space–time cluster analysis in SatScan (Scan Statistics, LLC., 2024) to examine the spatiotemporal patterns of isolation/loneliness-related conversations from 2016 to 2022. SatScan is a program developed to identify disease clusters in space, time, and space–time among heterogeneous population densities (Kulldorff, 2018). Similar to previous work, we utilized a discrete Poisson space–time scan statistic with a 10% at-risk population to identify localized clusters from January 1st, 2016, to December 31st, 2022 (Gao et al., 2014; Harden et al., 2021; Ryan et al., 2023; Sheehan et al., 2004). Our case data was the total number of isolation/loneliness conversations per area code per month, and our population data was the total number of crisis conversations per area code per month (Harden et al., 2021; Ryan et al., 2023). Max cluster length was set to 3 months after a preliminary analysis of 3, 6, 9, 12, and 18 months. Monte Carlo replications (n = 9999) were performed to assess the statistical significance of each cluster, with the null hypothesis being rejected at *p* < 0.05. Results also include a local relative risk (RR) for each area code within the study area as a measure of relative prevalence when comparing cluster area codes and times to non-cluster area codes and times. An RR > 1 indicates an increased prevalence within that area code compared to non-cluster area codes. Final clusters were identified and exported into ArcGIS Pro to map cluster locations and RR at the area code level (Esri, 2024).

### Proportional time series

A monthly time series was created in R, illustrating the proportion of isolation/loneliness conversations to all conversations that occurred by month from 2016 to the end of 2022 (R Core Team, 2024). A temporal stratum was included to represent March 11th, 2020, the day the Federal State of Emergency was declared for COVID-19.

Multiple summary demographic tables of our sample were generated in R using the *Tableone* package (Yoshida & Bartel, 2022), which employs a *chi*-square test to assess if demographic characteristics vary significantly among subgroups. We compared (1) Isolation/loneliness versus non-isolation/loneliness conversations among texters with demographic information, (2) Texters within and outside the identified isolation/loneliness clusters, and (3) texter demographics across the three COVID-19 periods. To further examine group differences in help-seeking, the Dunnett–Tukey–Kramer (DTK) Pairwise Multiple Comparison Test was run using the DTK package in R to account for unequal sample sizes and unequal variances among groups (Lau, 2013).

### Jaccard indices

We applied the Jaccard index (Real & Vargas, 1996) to understand the co-occurrence of other crisis topics in conversations related to isolation/loneliness. Data subsets were created for each crisis counselor post-conversation survey tags to assess the co-occurrence of the crisis topic. Data was also stratified by race and gender to understand better the demographic patterns of mental health outcomes associated with isolation/loneliness and by both age and the COVID-19 period to analyze how the pandemic affected the different age groups. Finally, we stratified by cluster membership to examine the effects of isolation/loneliness clusters. Cluster membership was determined by a conversation in an identified cluster area code and during the cluster time period.

## Results

### Descriptive statistics

From January 2016 to December 2022, there were 183,379 isolation/loneliness-related conversations for texters 24 years of age or younger (Table 1). Of conversations tagged with isolation/loneliness, LGBTQ+ (54.1%), girl/woman (73.6%), and transgender and gender diverse (13.7%) individuals were most prevalent, which is consistent with the demographics of all CTL conversations. The proportion of isolation/loneliness conversations by age, race, and ethnicity is similar to that of all CTL conversations. A series of supplemental Tukey’s post-hoc tests revealed significant differences between most of the age groups (Supplemental Figure 1), many of the racial identities (Supplemental Figure 2), both sexual orientation groups (Supplemental Figure 3), all three gender identity categories (Supplemental Figure 4).

**Table 1:** Demographic information from isolation/loneliness and non-isolation/loneliness conversation topics among individuals 24 and younger

	Isolation/loneliness conversation	Non-isolation/loneliness conversation	*p* value	SMD1
Total	183,379	792,258		
*Age*				
Total	183,379 (100.0%)	792,258 (100.0%)	< 0.001	0.021
13 or younger	33,637(18.3%)	149,583 (18.9%)		
14–17	84,153 (45.9%)	364,594 (46.0%)		
18–24	65,589 (35.8%)	278,081 (35.1%)		
*Race*				
Total	168,445 (91.9%)	733,595 (92.6%)	< 0.001	0.058
Asian and Pacific Islander2	8046(4.7%)	34,559 (4.7%)		
Black or African American	14,989 (8.9%)	58,713 (8.0%)		
Hispanic3	31,609 (18.8%)	130,204 (17.8%)		
Indigenous4	3735 (2.2%)	18,021 (2.5%)		
Other race and multiracial5	21,862 (13.0%)	91,942 (12.5%)		
White	88,204 (52.4%)	400,156 (54.5%)		
*Sexuality*				
Total	148,940 (81.2%)	655,535 (82.7%)	< 0.001	0.045
LGBTQ+7	80,595 (54.1%)	362,753 (55.3%)		
Straight or Heterosexual	68,345 (45.9%)	292,782 (44.7%)		
*Gender*				
Total	172,761 (94.2%)	750,525 (94.7%)	< 0.001	0.062
Girl/woman	127,157(73.6%)	557,435 (74.3%)		
Boy/man	21,902 (12.7%)	82,010 (10.9%)		
Transgender and gender diverse6	23,702 (13.7%)	111,080 (14.8%)		

1. Standardized mean differences (SMDs) communicate the effect of the variable, with an SMD of ~ 0.2 indicating marginal effect, ~ 0.5 indicating moderate effect, and ~ 0.8 indicating substantial effect (Cohen, 1988; Faraone, 2008) on identified demographic differences

2. Asian and Pacific Islander is an aggregated race category including Asian Americans, individuals from any region of Asia, and individuals who identified as Native Hawaiian or Pacific Islander

3. Hispanic includes Latine, Latino, Latina and Latinx identities

4. Indigenous Americans include Native Americans, Native Alaskans or Indigenous

5. Other race and multiracial is an aggregate category including individuals of other race, mixed race, and Middle Eastern, North African, or Arab descent

6. Transgender and gender diverse (TGD) is an aggregate category of texters who identify as trans, genderqueer, agender, intersex, other gender, genderfluid, transmasc, transfem, non-binary, and two-spirit identities

7. LGBTQ+ includes asexual, aromantic, bisexual, gay, lesbian, pansexual, queer, questioning and not sure sexualities

### Spatiotemporal analysis

**Fig. 1:**
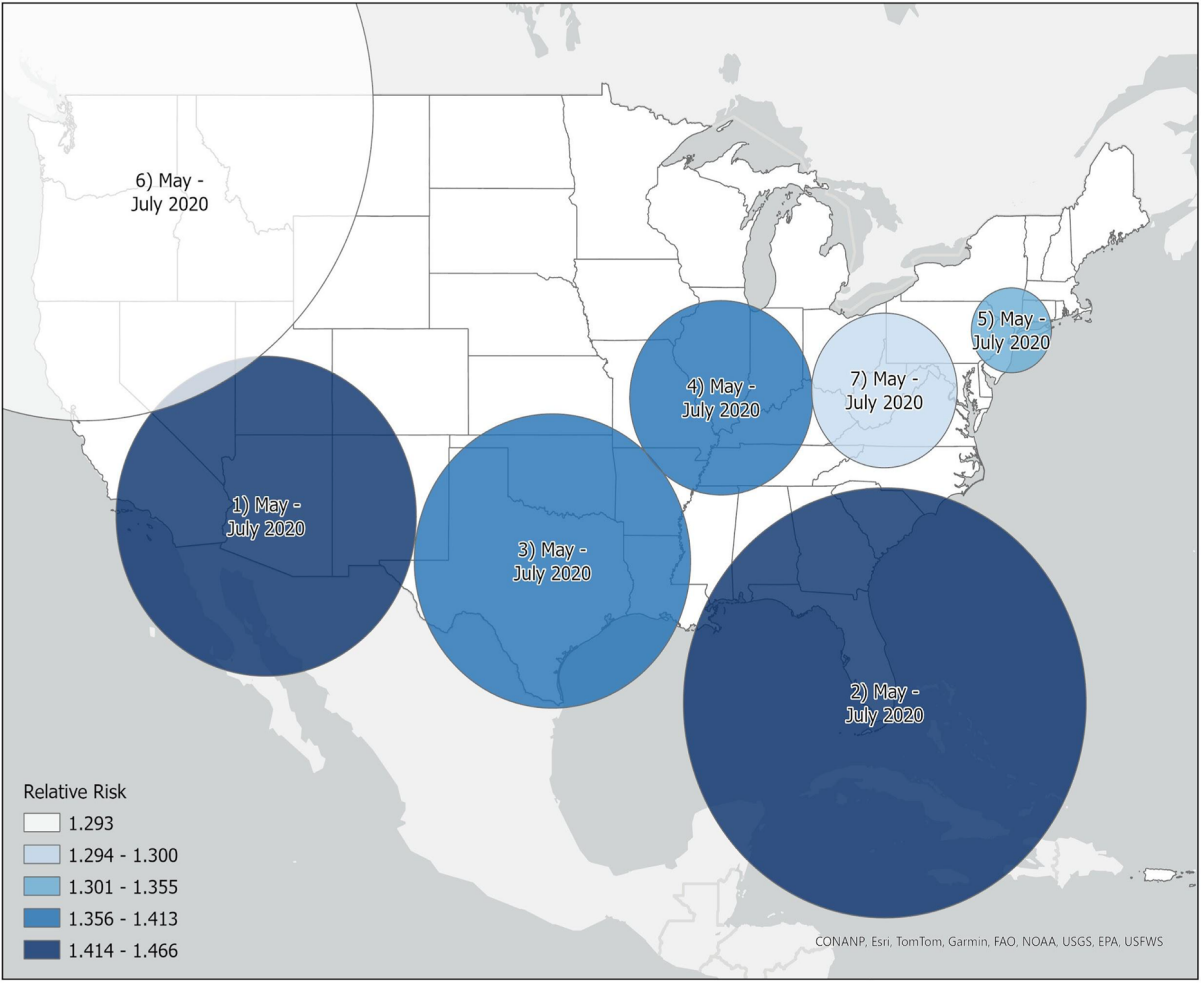
Spatial distribution of isolation/loneliness clusters labeled with their likelihood (1 = most likely) and the temporal prevalence. Relative risk (RR) values of 1 = average risk, RR > 1 = greater risk, and RR < 1 = lesser risk

**Fig. 2:**
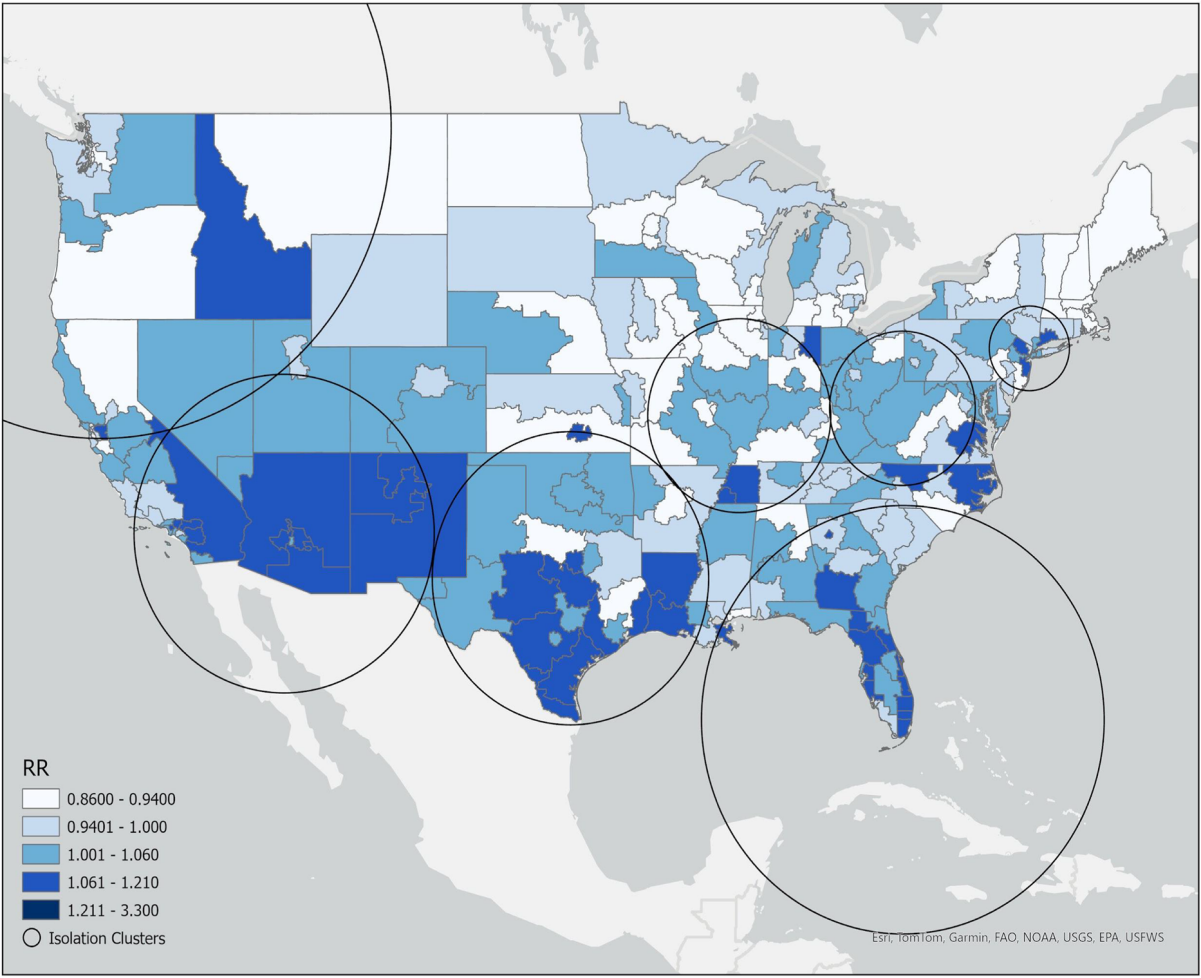
Spatial distribution of relative risk (RR) values for each area code in the United States. Nearly all area codes with an elevated RR value > 1.06 occur within clusters

Spatiotemporal cluster analysis via SatScan resulted in the identification of seven significant (*p* < 0.05) isolation/loneliness clusters with elevated relative risks (RRs) between 1.29 and 1.47 (Fig. 1). All identified clusters occurred from May 2020 through July 2020, with the highest likelihood cluster (log-likelihood: *p* < 0.001) identified in the American Southwest. Secondary clusters (RR: 1.30 to 1.47) were identified throughout the Sunbelt region and in the Pacific Northwest, Great Lakes, and New York (Fig. 2).

**Fig. 3:**
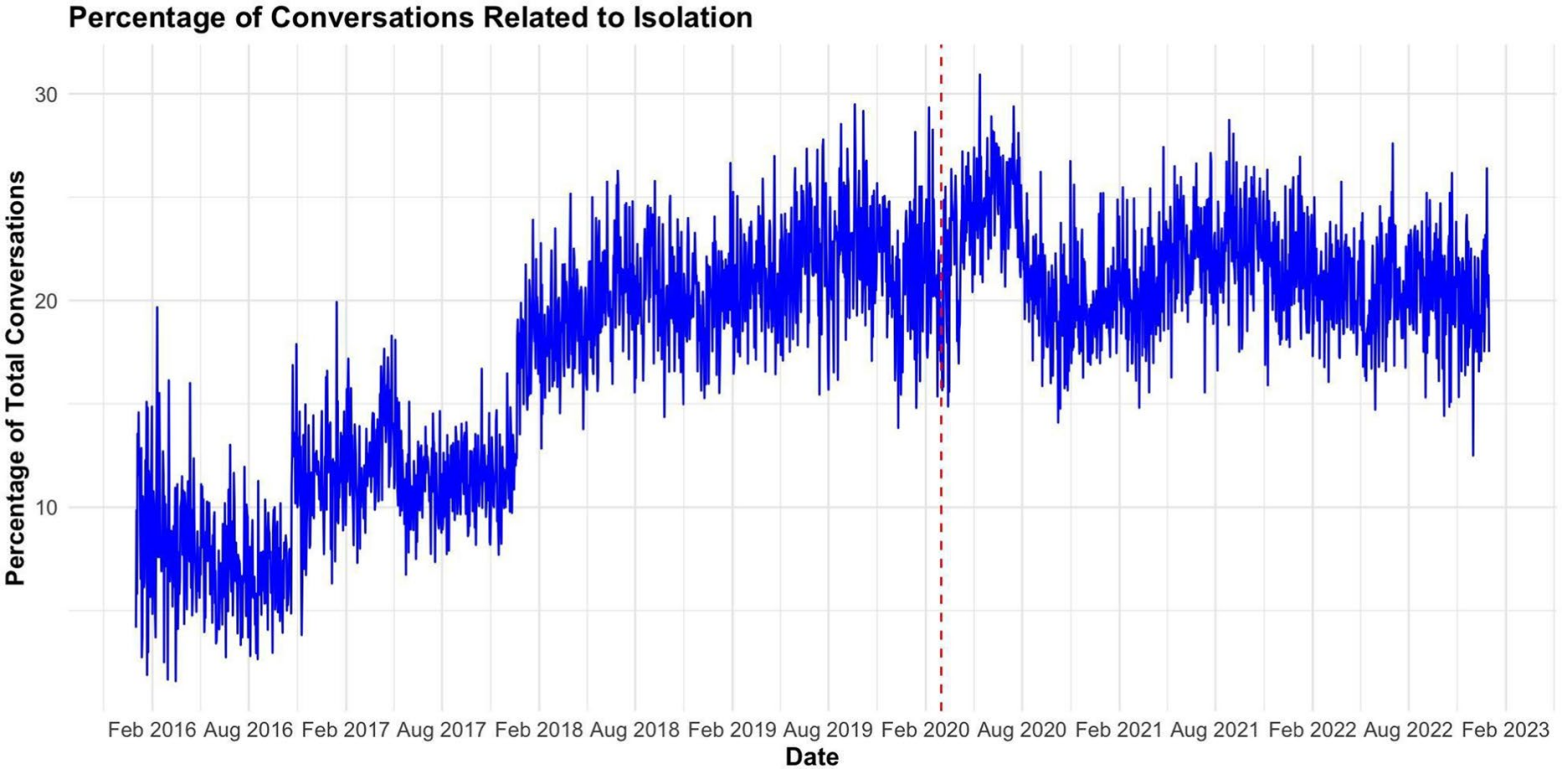
Time-series of the proportion of isolation/loneliness-related conversations in relation to all CTL conversations aggregated by month. The red dashed line represents March 11th, 2020, when the state of emergency was declared for COVID-19. The proportion of isolation/loneliness conversations increases in the months following the declaration of the state of emergency, peaking at over 30%

A temporal analysis was conducted via a proportional time-series (Fig. 3), which visualizes the percentage of daily conversations relating to isolation/loneliness within the entire CTL dataset. The red dashed line represents March 11th, 2020, when the state of emergency was declared for COVID-19. There was a spike in the percentage of users experiencing isolation/loneliness (20–25% to 25–30%) beginning in March 2020, with the proportion of isolation/loneliness conversations peaking at over 30% in the spring of 2020 (Fig. 3). The Early COVID-19 period, despite constituting the least amount of time (13 months), averaged over 3,196 isolation conversations per month (41,559 conversations/13 months), significantly higher compared to the pre-COVID-19 (1865 conversations/month) and the late-COVID-19 periods (2964 conversations/month).

To further explore these spatiotemporal patterns of isolation/loneliness, a demographics table was generated for isolation/loneliness conversations with a strata of identified cluster membership (Table 2). The most significant differences in this comparison were White youth (− 8.1%), 18 to 24-year-olds (− 3.9%), and transgender and gender-diverse youth (− 3.2%), who were less prevalent in the identified clusters.

**Table 2:** Demographics table comparing isolation/loneliness conversations that occurred within a cluster to outside of them

	Cluster	Non-cluster	*p* value	SMD
	8388	174,991		
*Age*				
Total	8388 (100.0%)	174,991 (100.0%)	< 0.001	0.292
13 or younger	1817 (21.7%)	31,820(18.2%)		
14–17	3889 (46.4%)	80,264 (45.9%)		
18–24	2682 (32.0%)	62,907 (35.9%)		
*Race*				
Total	7884 (94.0%)	160,561 (91.8%)	< 0.001	0.207
Asian and Pacific Islander	491 (6.2%)	7555 (4.7%)		
Black or African American	756 (9.6%)	14,233 (8.9%)		
Hispanic	1935 (24.5%)	29,674 (18.5%)		
Indigenous	140 (1.8%)	3595 (2.2%)		
Other race and multiracial	1049 (13.3%)	20,813 (13.0%)		
White	3513 (44.6%)	84,691 (52.7%)		
*Sexuality*				
Total	7479 (89.2%)	141,461 (80.8%)	< 0.001	0.235
LGBTQ+	3991 (53.4%)	76,604 (54.2%)		
Straight	3488 (46.6%)	64,857 (45.8%)		
*Gender*				
Total	8084 (96.4%)	164,677 (94.1%)	< 0.001	0.175
Girls/women	6389 (79.0%)	120,768 (69.0%)		
Boys/men	893 (11.1%)	21,009 (12.0%)		
Transgender and gender diverse	802 (9.9%)	22,900 (13.1%)		

Standardized mean differences (SMDs)1 communicate the effect of the variable, with an SMD of ~ 0.2 indicating marginal effect, ~ 0.5 indicating moderate effect, and ~ 0.8 indicating substantial effect (Cohen, 1988; Faraone, 2008) on identified demographic differences

The Jaccard Similarity Indices indicate that depression/sadness (51.3%), relationship problems (47.2%), anxiety (34.1%), and suicide (27.9%) tags were the most likely to be identified alongside isolation/loneliness within clusters. Interestingly, co-occurrence rates diminish slightly within clusters versus outside of clusters, especially for thoughts of suicide. However, this could be caused by the disproportionate amount of isolation/loneliness conversations within clusters (25.8%) compared to non-cluster locations (18.6%) (Table 3).

**Table 3:** Clusters Jaccard Similarity Index of rates of co-occurrence between isolation/loneliness and other mental health outcomes in isolation/loneliness spatiotemporal clusters and non-spatiotemporal clusters

	Cluster	Non-cluster
Depression/sadness	0.513	0.516
Anxiety/stress	0.341	0.355
Suicide	0.279	0.333
Self harm	0.148	0.164
Substance use	0.016	0.021
Relationship	0.472	0.483

### Co-occurrence of crisis events among race and gender groups

Results from the racially stratified Jaccard Similarity Index (Table 4) demonstrated that Indigenous youth had the highest co-occurrence between isolation/loneliness and substance use (2.4%), self-harm (18.8%), and suicide (37.3%) of any racial demographic. Asian Americans and Pacific Islander youth had the highest co-occurrence between isolation/loneliness and both anxiety/stress (39.3%) and relationship (50.7%) tags, and Hispanic youth had the highest co-occurrence of isolation/loneliness and depression/sadness (52.1%).

**Table 4:** Race Jaccard Similarity Index for rates of co-occurrence between isolation and other crisis text tags among six different race categories

	White	Black or African American	Hispanic	Indigenous American	Asian and Pacific Islander	Middle Eastern and mixed/other
Depression/sadness	0.516	0.519	0.521	0.516	0.5	0.515
Anxiety/stress	0.358	0.326	0.353	0.348	0.393	0.36
Suicide	0.333	0.333	0.315	0.374	0.316	0.337
Self harm	0.173	0.127	0.154	0.188	0.122	0.154
Substance use	0.022	0.015	0.021	0.024	0.017	0.019
Relationship	0.479	0.462	0.5	0.493	0.507	0.485

The Jaccard Similarity methodology was also utilized to stratify the data by gender (Table 5). Based on these descriptive statistics, transgender and gender-diverse youth had the highest co-occurrence rates between isolation/loneliness and anxiety/stress (36.5%), suicide (39.6%), and self-harm (21.1%) (Table 5).

**Table 5:** Gender Jaccard Similarity Index for rates of co-occurrence between isolation and other crisis text tags compared between 3 genders

	Nonconforming	Male	Girls/women
Depression/sadness	0.512	0.519	0.517
Anxiety/stress	0.365	0.329	0.358
Suicide	0.396	0.344	0.315
Self harm	0.211	0.104	0.161
Substance use	0.023	0.032	0.018
Relationship	0.464	0.483	0.487

Results from the methodology using the Jaccard Similarity Index, in which we stratified the data into three different time periods and age groups (Supplemental Table 1; Supplemental Table 2), revealed that depression/sadness (53.4%), relationship problems (51.1%), anxiety/stress (40.2%), and suicide (39%) were the outcomes with the highest rates of co-occurrence. However, these rates were not uniform across the age groups and time periods, such that thoughts of suicide and self-harm had the highest rates of co-occurrence within the 13 or younger age group during the pre-COVID-19 and Early COVID-19 periods. Furthermore, 18 to 24-year-olds had higher overall rates of co-occurrence for depression/sadness (early COVID-19) and anxiety/stress (late COVID-19) than any other age group. Other findings include the overall decreasing co-occurrence of depression/sadness and relationship issues as well as increasing anxiety and substance use among all ages. The specific direction of these mental health trends varies based on the outcome and age group. For all age-based trends, the youngest (13 and younger) and oldest (18–24) age groups constitute the extremes, with the 14 to 17-year-olds somewhere between. For example, in the suicide tag, individuals 13 or younger noted a co-occurrence rate of 39% Pre-COVID-19, 14 to 17-year-olds had a co-occurrence rate of 34.9% Pre-COVID-19, and 18 to 24-year-olds had a co-occurrence rate of 33.9% Pre-COVID-19.

## Discussion

Our study extends the knowledge base by documenting significant increases in the isolation and loneliness burden among youth and young adults following the onset of the COVID-19 pandemic. The adherence to social distancing, working and schooling at home, and reduced time with friends and family significantly disrupted daily lives, strained support systems, and resulted in many young people feeling isolated (Bell et al., 2023; Cohen & Bosk, 2020). We provide some of the first evidence of spatial clustering of isolation/loneliness during the COVID-19 pandemic, identifying 7 spatiotemporal clusters of isolation/loneliness, all occurring from May to July 2020. Further analysis of the relationships between isolation/loneliness and other crises of concern found the highest correlations between isolation and depression/sadness, relationship problems, anxiety, and suicide.

Geographically, we found that the most severe increases in isolation/loneliness occurred primarily in the US Sunbelt region. This region, which includes part or the whole of North Carolina, Georgia, Alabama, Mississippi, Tennessee, Louisiana, Arkansas, Oklahoma, Texas, New Mexico, Arizona, Florida, Nevada, and Southern California (Strom, 2017), had the three highest risk clusters, with secondary clusters identified in the Pacific Northwest, Midwest, and Northeast. While these regions are culturally, economically, and socially different from one another, they all had elevated levels of COVID-19 cases early in the pandemic compared to the rest of the country (Andersen et al., 2021). Studies on COVID-19 transmission and hotspots with temporal overlap with our identified clusters consistently identify these regions as having high case counts (Andersen et al., 2021; Oster et al., 2020). The spatiotemporal overlap in COVID-19 clusters and hotspots with isolation clusters suggests a strong association with COVID-19 outbreaks and feelings of isolation among youth.

The effects of the COVID-19 pandemic on isolation were not demographically uniform, with several racial minorities experiencing a disproportionately heightened mental health burden. Cluster locations reflected an increased prevalence of Hispanic, Asian and Pacific Islanders and Black or African American youth when compared to non cluster locations. This finding is consistent with the Surgeon General’s 2021 advisory that stated that Black or African American, Hispanic, Indigenous, Asian and Pacific Islander youth and young adults were at higher risk of mental health challenges during the pandemic due to a wide range of additional stressors (Office of the Surgeon General, 2021), including a dispropriate impact on deaths (Garcia et al., 2021), additional financial stresses and limited social support (Thomeer et al., 2022) and discrimination (Strassle et al., 2022).

COVID-19-induced isolation affected the entire country, but these data indicate it was especially impactful on Indigenous and Black or African American populations who faced additional challenges with heightened cases and deaths as well as obstacles in staying connected with loved ones within Indigenous communities. The biggest obstacle to connection in Indigenous communities was the limited internet access (Federal Communications Commission; 2019). In addition to limited internet access, heightened COVID-19 case and death counts within Indigenous and African American communities led to increases in young adult bereavement (Harden et al., 2021), which is associated with heightened suicide risk (Molina et al., 2019; Hamdan et al., 2020; Stroebe et al., 2005). Corroborating these findings, we found that Indigenous and Black or African American youth were consistently identified as having two of the top three highest co-occurrence rates between isolation/loneliness and suicide and depression/sadness tags. The heightened cases and deaths associated with COVID-19, in addition to limited internet access in Indigenous communities, likely led to increased isolation.

Girls/women and transgender and gender-diverse individuals seek help at higher rates than boys/men. This finding is consistent with the literature, indicating women are more likely to seek help and to recognize the need for help (Ang et al., 2004; Morgan et al., 2003; Perenc & Radochonski, 2016). Results also revealed that among those who texted about isolation/loneliness, transgender and gender-diverse individuals were more likely to have co-occurring anxiety, substance use, self-harm, and suicidal thoughts compared to girls/women only and men/boys only.

Our findings on isolation patterns are important because research has shown a significant association between the experience of isolation and the development of suicidality, which is most prevalent among younger age groups. According to the Interpersonal Theory of Suicide, social isolation is the “strongest and most reliable predictor” of suicidal thoughts and actions (Van Orden et al., 2010). Results of our study indicate that the age group with the highest co-occurrence between isolation and every suicide metric was the youngest age group, individuals 13 or younger. Individuals aged 18 to 24 years old had the lowest co-occurrence between isolation and suicide metrics, which is consistent with other age-based trends identified in this study. These results suggest that the younger an individual is when they seek help for isolation, the more likely they are to be experiencing some degree of suicidality. Our results are also noteworthy as clusters of isolation are also most found among the youngest age group.

The spatio-temporal clusters presented in this study demonstrate that substantial community-level threats, such as the COVID-19 pandemic, can contribute to increased rates of isolation/loneliness among young people within that community. This geographical approach to the study of isolation/loneliness is relatively uncommon in the literature, which tends to study isolation/loneliness on an individual-level. Future research in this field would benefit greatly from considering a geographical approach, which can aid in the identification of community-level factors impacting the mental health of young people. This allows for community leaders to implement community interventions aimed to reduce the severity of factors contributing to elevated levels of isolation/loneliness in the area, and bolstering resources that could protect against their psychological impact. By limiting the geographic scale of the study area, researchers can be more specific as to which areas are disproportionately impacted and more accurately identify correlated factors among different populations experiencing heightened isolation/loneliness.

While the exact strength and direction of the relationship between isolation/loneliness and COVID-19 outbreaks was not explored in this study, results from previous literature suggests that clustering of isolation conversations in the midst of COVID-19 outbreaks could be related to the relative fear of COVID-19 experienced by youth in those areas. A cross-national analysis of isolation and psychological distress in 2020 noted that the isolation-distress connection is greater in countries with more coronavirus-induced deaths and less strict policy measures (Kim & Jung, 2020). Furthermore, we noted visual similarities between isolation/loneliness clusters and communities with high levels of social vulnerability. Social vulnerability factors, including poverty, housing characteristics, and minority population, have been shown to be strong predictors of a community’s ability to respond to hazards and stressors (Flanagan et al., 2018; Juntunen, 2005). The CDC’s Social Vulnerability Index is an overall measure of the demographic and socioeconomic factors of a community that can affect its ability to respond to a community-level threat (Centers for Disease Control and Prevention, 2024). The SVI has been shown to be a strong predictive factor for assessing a community’s COVID vulnerability in predictive models due to the facilitation of COVID transmission across avenues of social vulnerability (Dasgupta et al., 2020; Huang et al., 2022; Tatar et al., 2023). We noted similar spatial patterns when examining our isolation RRs, the SVI, and COVID-19 clusters identified by Harden et al. and Ryan et al. (Harden et al., 2021; Ryan et al., 2023). Though we did not include SVI in our analysis, these results suggests that social vulnerability may have influenced the differential impacts of COVID-19 transmission, increasing cases, deaths, and isolation.

While there have been many previous studies on the differentiation in isolation/loneliness among different ages, exploratory findings presented in this study indicate there are also differences in the effects of the COVID-19 pandemic on loneliness/isolation among different racial, gender, and sexual orientation identities as well. Given the exploratory nature of these results, further research is needed to draw more concrete correlations between isolation/loneliness and different racial, gender, and age groups.

This study does have a few limitations. For the spatiotemporal analysis, the CTL dataset was aggregated to the area code level, which provides a level of geographical coarseness that does not allow for causal associations to be drawn based on differences based on race, gender, ethnicity, or rural vs. urban populations, which are all available at finer spatial scales. However, this remains the first study within the United States to identify subnational isolation patterns. The repository examined may not represent all CTL users or the general US adolescent population. It may be subject to nonrandom selection bias toward individuals who provided their age in the demographic survey. Territories and states not in the contiguous US were excluded from this study, so we could not capture any potential spatiotemporal trends within these populations. Future research would benefit from examining these trends at a finer spatial scale to draw more detailed conclusions on community-wide characteristics and vulnerabilities.

## Conclusions

Our study extends current literature on the mental health impacts of COVID-19 on youth and young adults through an analysis of the spatiotemporal patterns of isolation from 2016 to 2022. Findings suggest that the COVID-19 pandemic increased the prevalence of isolation, especially in the Sunbelt from May to July 2020. Analysis of demographic characteristics identified the disproportionate prevalence of isolation/loneliness among texters 13 and younger, girls/women, minorities, and LGBTQ+ individuals compared to the overall CTL population. Isolation/loneliness was found to most often co-occur with depression/sadness, relationship issues, anxiety, and suicide. The regions identified within clusters can guide the mobilization of mental health resources in future national crises, resulting in loss of life and significant disruptions to daily life.

## Supplementary Information

Below is the link to the electronic supplementary material.

Supplementary file1 (DOCX 153 kb)
