# Supplementary material for: Spatiotemporal patterns of youth isolation and loneliness in the US: a geospatial analysis of Crisis Text Line data (2016–2022)
Source: GeoJournal. 2024 Dec 3;89(6):249. doi: 10.1007/s10708-024-11253-w (PMC11614998; doi:10.1007/s10708-024-11253-w)
Supplement: Supplementary file 1 — Supplementary file1 (DOCX 153 kb) [file 10708_2024_11253_MOESM1_ESM.docx]

**Supplemental Material**


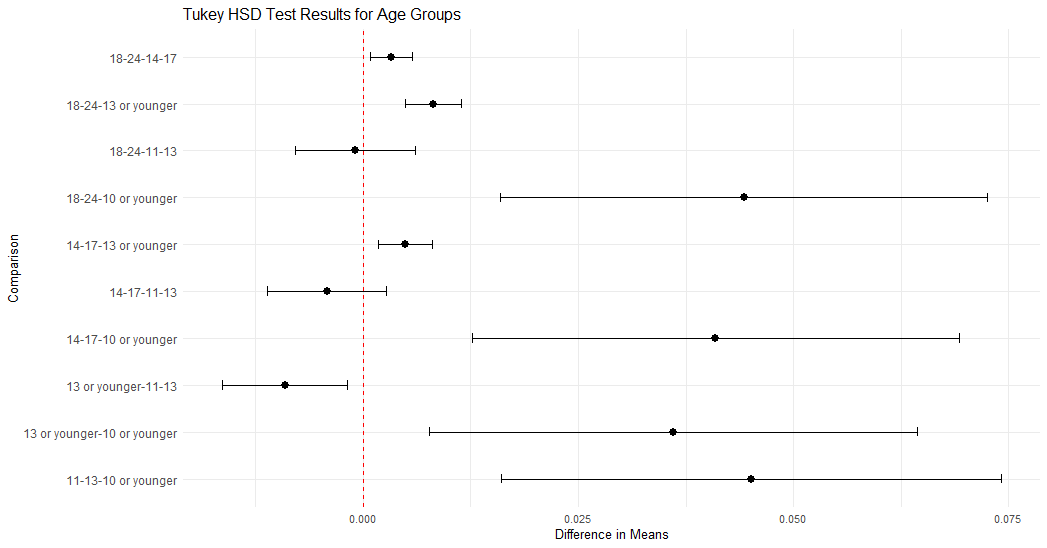


**Supplemental Figure 1**. Pairwise comparisons of age groups for isolation conversations, based on Tukey's Honest Significant Difference (HSD) post-hoc test. The plot displays the mean differences between groups along with 95% confidence intervals. A dashed red line at y = 0 indicates no difference between groups. Comparisons whose confidence intervals do not overlap with zero represent statistically significant differences in the likelihood of isolation conversations between the respective groups.


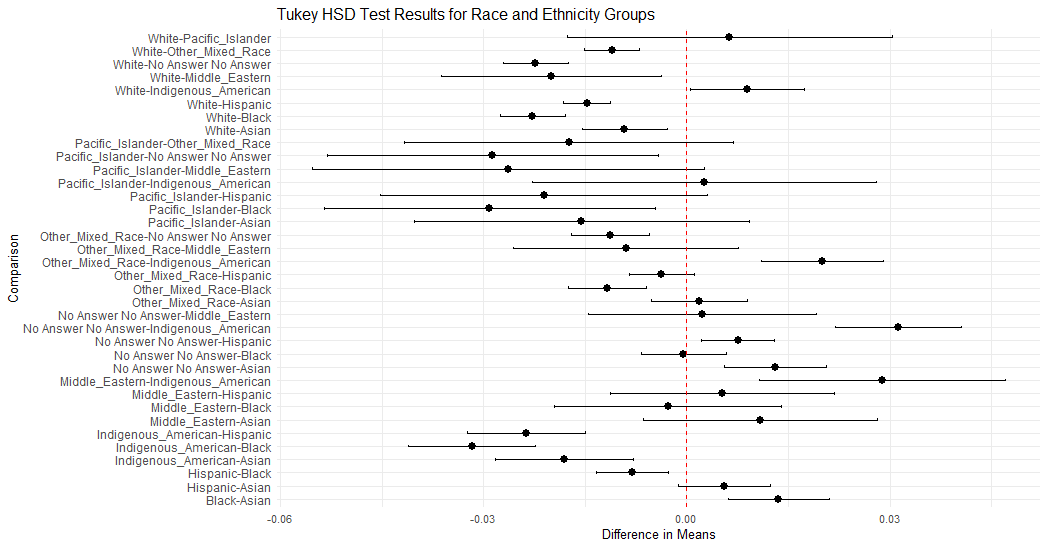


**Supplemental Figure 2.** Pairwise comparisons of race and ethnicity groups with respect to isolation conversations, based on Tukey's Honest Significant Difference (HSD) post-hoc test. The plot displays the mean differences between groups along with 95% confidence intervals. A dashed red line at y = 0 indicates no difference between groups. Comparisons whose confidence intervals do not overlap with zero represent statistically significant differences in the likelihood of isolation conversations between the respective groups.


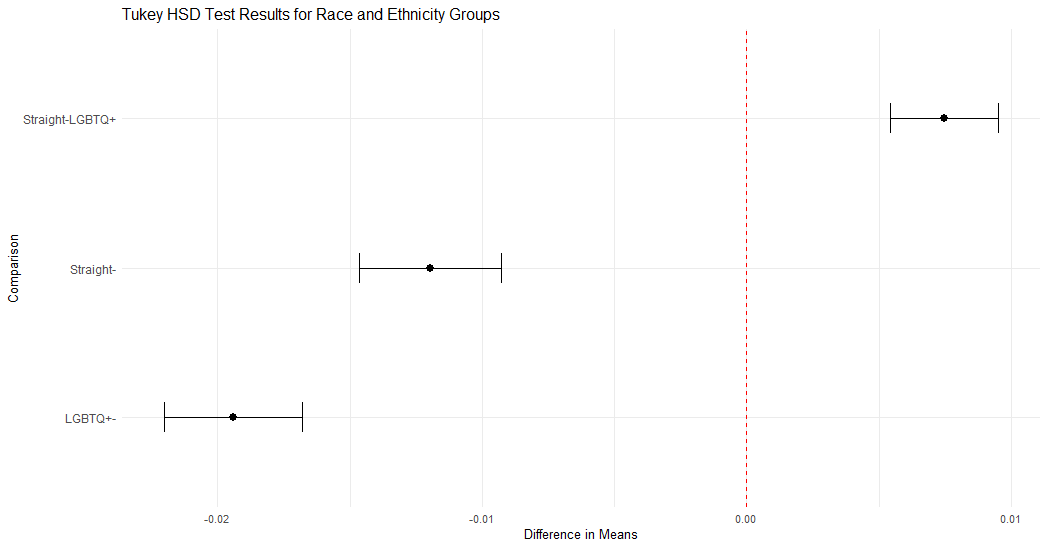


**Supplemental Figure 3**. Pairwise comparisons of sexuality groups with respect to isolation conversations, based on Tukey's Honest Significant Difference (HSD) post-hoc test. The plot displays the mean differences between groups along with 95% confidence intervals. A dashed red line at y = 0 indicates no difference between groups. Comparisons whose confidence intervals do not overlap with zero represent statistically significant differences in the likelihood of isolation conversations between the respective groups.


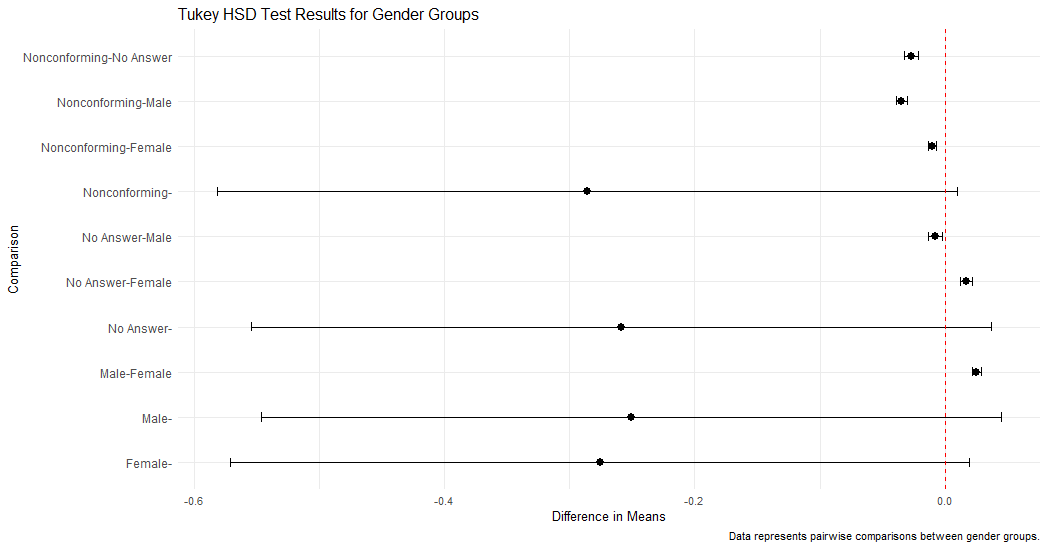


**Supplemental Figure 4**. Pairwise comparisons of sexuality groups with respect to isolation conversations, based on Tukey's Honest Significant Difference (HSD) post-hoc test. The plot displays the mean differences between groups along with 95% confidence intervals. A dashed red line at y = 0 indicates no difference between groups. Comparisons whose confidence intervals do not overlap with zero represent statistically significant differences in the likelihood of isolation conversations between the respective groups.

Supplemental Table 1: Demographics table comparing the pre-COVID-19 time period to the COVID-19 time period.

|  |  | **PreCOVID-19 Isolation Conversations** | **Early COVID-19 Isolation Conversation** | **Late COVID-19 Isolation Conversation** | **p** | **SMD** |
| --- | --- | --- | --- | --- | --- | --- |
|  |  | 91420 | 41559 | 50400 |  |  |
|  |  |  |  |  |  |  |
| **Age** | Total | 91420 (100.0%) | 41559 (100.0%) | 50400 (100.0%) | <0.001 | 0.369 |
|  | 13 or younger | 14962 (16.4%) | 8281 (19.9%) | 10395 (20.6%) |  |  |
|  | 14-17 | 43441 (47.5%) | 19007 (45.7%) | 21705 (43.1%) |  |  |
|  | 18-24 | 33017 (36.1%) | 14271 (34.3%) | 18301 (36.3%) |  |  |
|  |  |  |  |  |  |  |
| **Race** | Total | 87350 (95.5%) | 39190 (94.3%) | 41905 (81.5%) |  |  |
|  | Asian and Pacific Islander | 4546 (5.2%) | 2284 (5.8%) | 1216 (2.9%) |  |  |
|  | Black or African American | 7023 (8.0%) | 3622 (9.2%) | 4344 (10.4%) |  |  |
|  | Hispanic | 15731 (18.0%) | 7900 (20.2%) | 7978 (19.0%) |  |  |
|  | Indigenous | 2083 (2.4%) | 773 (2.0%) | 879 (2.1%) |  |  |
|  | Other Race and Multiracial | 10673 (12.2%) | 5183 (13.2%) | 6006 (14.4%) |  |  |
|  | White | 47294 (54.2%) | 19428 (49.6%) | 21482 (51.3%) |  |  |
|  |  |  |  |  |  |  |
| **Sexuality** | Total | 84114 (92.0%) | 37522 (90.3%) | 27304 (54.2%) | <0.001 | 0.653 |
|  | LGBTQ+ | 42935 (51.0%) | 20744 (55.3%) | 16916 (62.0%) |  |  |
|  | Straight | 41179 (49.0%) | 16778 (44.7%) | 10388 (38.0%) |  |  |
|  |  |  |  |  |  |  |
| **Gender** | Total | 88712 (97.0%) | 40203 (96.7%) | 43846 (87.0%) | <0.001 | 0.314 |
|  | Girl/Woman | 67121 (75.7%) | 30758 (76.5%) | 29278 (66.8%) |  |  |
|  | Boy/Man | 11612 (13.1%) | 4573 (11.4%) | 5717 (13.0%) |  |  |
|  | Transgender and Gender Diverse | 9979 (11.2%) | 4872 (12.1%) | 8851 (20.2%) |  |  |

Supplemental Table 2: Age/COVID-19 Jaccard Similarity Index. This figure demonstrates the rates of co-occurrence between isolation and several other mental health outcomes compared along 3 different age groups and stages of the COVID-19 pandemic.

|  | 13 or Younger | | | 14 to 17 Years Old | | | 18 to 24 Years Old | | |
| --- | --- | --- | --- | --- | --- | --- | --- | --- | --- |
| COVID-19 Stage | Pre- COVID-19 | Early COVID-19 | Late COVID-19 | Pre- COVID-19 | Early COVID-19 | Late COVID-19 | Pre- COVID-19 | Early COVID-19 | Late COVID-19 |
| depression/sadness | 0.495 | 0.505 | 0.465 | 0.514 | 0.528 | 0.497 | 0.534 | 0.531 | 0.529 |
| Anxiety | 0.301 | 0.320 | 0.341 | 0.331 | 0.356 | 0.375 | 0.366 | 0.401 | 0.402 |
| Suicide | 0.390 | 0.353 | 0.363 | 0.349 | 0.310 | 0.307 | 0.339 | 0.279 | 0.288 |
| Self Harm | 0.205 | 0.202 | 0.231 | 0.162 | 0.163 | 0.197 | 0.129 | 0.125 | 0.128 |
| Substance Use | 0.010 | 0.013 | 0.021 | 0.016 | 0.019 | 0.027 | 0.024 | 0.026 | 0.031 |
| Relationship | 0.502 | 0.462 | 0.478 | 0.511 | 0.464 | 0.471 | 0.489 | 0.448 | 0.456 |
